# Editorial: Cognitive reserve, cognitive functioning, and mental health in elderly people

**DOI:** 10.3389/fnhum.2022.1040675

**Published:** 2022-10-04

**Authors:** Tatiana Quarti Irigaray, Carmen Moret-Tatay, Mike Murphy, Camila Rosa de Oliveira

**Affiliations:** ^1^Pós-Graduate Program in Psychology, Pontifícia Universidade Católica Do Rio Grande Do Sul, Porto Alegre, Brazil; ^2^Faculty of Psychology, Universidad Católica de Valencia San Vicente Mártir, Valencia, Spain; ^3^School of Applied Psychology, University College Cork, Cork, Ireland; ^4^Pós-Graduate Program in Psychology, IMED, Passo Fundo, Brazil

**Keywords:** cognitive reserve, aging, mental health, cognitive functioning, elderly

Biological, psychological, and social changes are features of the entire lifespan. These three dimensions have typically been employed in distinguishing between successful, pathological, and normal aging (Rowe and Kahn, 1987). Understanding this classification and its underlying variables is important in promotion of quality-of-life at all ages (Stewart et al., 2019); for example one should bear in mind that impairments in mental and physical health, and deficits in cognition of older adults, might occur, altering brain structure and cognitive processing and potentially leading to mild cognitive impairment and even dementia. Latterly, the scientific community has increased its focus on variables, both internal and external, that might slow down the progression of disease or impairment in the elderly.

The term “cognitive reserve” refers to cognitive or mental tolerance to physiological brain changes related to age or pathology (Mondini et al., 2016). Different mechanisms for explaining this phenomenon—such as brain reserve, cognitive reserve, and brain maintenance—have been advanced (Stern et al., 2020). In relation to mental health, previous literature has found that poorer cognitive performance is positively associated with anxiety, as well as negatively associated with cognitive reserve (Farina et al., 2021; García-Moreno et al., 2021). Considering that the relationship between these variables seems to be even more complex across the lifespan, this Research Topic aims to explore variables of interest in the field.

First, when deficits occur, one crucial issue is treatment adherence, which might be even more complicated when chronic conditions occur (Cho et al., 2018). A good and very prevalent example is chronic pain. According to a recent review, chronic pain affects a large number of older adults with myriad factors that influence each other, including affective dimensions (Dagnino and Campos). Pain can increase memory loss in Alzheimer's or motor symptoms in Parkinson's disease. Moreover, symptoms of anxiety and depression in elderly people with HIV-associated neurocognitive disorder, have been found to be associated with worse outcomes for processing speed, visual memory, attentional alternation, and visual perception (Barroso and Sousa). It is also noteworthy that pathological levels of anxiety and depression were found in the sample, highlighting how relevant it is to consider both cognitive and emotional dimensions from a health care perspective.

Secondly, innovative tools to measure cognition in the elderly are suggested by Brauner et al., bearing in mind that: (i) performing activities of daily living seems to be one of the most commonly addressed variables of interest in older adults (with a view to supporting independence in day-to-day life); and (ii) life activities involve different levels of cognitive demands. The authors examined the accuracy of cognitive responses as a contributing factor in estimating cost in a dual-task exercise in this context (Brauner et al.), and proposed a new index (P-index) which was correlated with the different Mini-Mental State Examination (MMSE) domains. This accuracy index seems to better reflect the variation of the MMSE compared to traditional time or speed approaches.

While the COVID-19 outbreak has triggering symptoms such as anxiety, stress and depression (Esteves et al., 2021), digital technology adoption might be an opportunity to address this at a population level, including for older adults (Oliveira et al., 2016; Moret-Tatay and Murphy, 2019). One example that might illustrate the benefits of Information and Communication Technology (ICT) adoption by the elderly is the scenario that COVID-19 outbreak has provided us. Romanopoulou et al. explored the impact of cognitive training intervention on the wellbeing of older adults and of vulnerable groups by employing an interactive web-based software. Results were of interest in promoting social inclusion and assistance of the older people, as these indicated an improvement in wellbeing as well as a decreased subjective distress caused by the traumatic COVID-19 related consequences.

Working from a cognitive impairment perspective, Cao et al. examined morphometric alterations in the cortical and subcortical structures in multiple system atrophy (MSA) patients with mild cognitive impairment (MCI), also studying the association with cognitive deficits. The authors suggest that cognitive decline due to temporal region alterations might not only be a characteristic of MSA-MCI patients, but also they describe potential biomarkers. In this context, another profile of interest is ischemic leukoaraiosis (ILA), which is related to cognitive impairment and vascular dementia in the elderly. By using DTI tractography and network analysis, Lu et al. found that disrupted white matter integrity and topological alterations of white matter networks in ILA was present in this population (Lu et al.). Moreover, these disturbances seem to be related to the severity of cognitive impairment.

In sum, these studies suggest that the affective dimension should not be seen as separate from the cognitive one in the assessment of mental health and functioning in older adults. They also underline an urgent need for innovation in screening techniques, as well as the study of biomarkers through neuroimaging techniques. Lastly, the adoption of digital technology is an opportunity for different age groups to remain independent and, ultimately, a variable of interest in the field of cognitive reserve. These results are of interest from both applied and theoretical levels. While years have been added to life through the increase in life expectancy, there is a worldwide concern to add life to years by developing strategies to prevent physical and mental illness in older adults, promoting quality of life. From a theoretical view, findings may be of interest in implementing current theories based on cognitive reserve.

## Author contributions

All authors listed have made a substantial, direct, and intellectual contribution to the work and approved it for publication.

## Conflict of interest

The authors declare that the research was conducted in the absence of any commercial or financial relationships that could be construed as a potential conflict of interest.

## Publisher's note

All claims expressed in this article are solely those of the authors and do not necessarily represent those of their affiliated organizations, or those of the publisher, the editors and the reviewers. Any product that may be evaluated in this article, or claim that may be made by its manufacturer, is not guaranteed or endorsed by the publisher.
